# Transcription Factor FoxO1 Is Essential for Enamel Biomineralization

**DOI:** 10.1371/journal.pone.0030357

**Published:** 2012-01-24

**Authors:** Ross A. Poché, Ramaswamy Sharma, Monica D. Garcia, Aya M. Wada, Mark J. Nolte, Ryan S. Udan, Ji-Hye Paik, Ronald A. DePinho, John D. Bartlett, Mary E. Dickinson

**Affiliations:** 1 Department of Molecular Physiology and Biophysics, Baylor College of Medicine, Houston, Texas, United States of America; 2 Program in Developmental Biology, Baylor College of Medicine, Houston, Texas, United States of America; 3 Department of Cytokine Biology, Forsyth Institute, and Department of Developmental Biology, Harvard School of Dental Medicine, Cambridge, Massachusetts, United States of America; 4 Department of Genetics, University of Texas M. D. Anderson Cancer Center, Houston, Texas, United States of America; 5 Department of Pathology and Laboratory Medicine, Weill Cornell Medical College, New York, New York, United States of America; 6 Departments of Medical Oncology, Medicine, and Genetics, Belfer Institute for Applied Cancer Science, Dana-Farber Cancer Institute and Harvard Medical School, Boston, Massachusetts, United States of America; University of Texas Health Science Center at Houston, United States of America

## Abstract

The Transforming growth factor β (Tgf-β) pathway, by signaling via the activation of Smad transcription factors, induces the expression of many diverse downstream target genes thereby regulating a vast array of cellular events essential for proper development and homeostasis. In order for a specific cell type to properly interpret the Tgf-β signal and elicit a specific cellular response, cell-specific transcriptional co-factors often cooperate with the Smads to activate a discrete set of genes in the appropriate temporal and spatial manner. Here, via a conditional knockout approach, we show that mice mutant for Forkhead Box O transcription factor *FoxO1* exhibit an enamel hypomaturation defect which phenocopies that of the *Smad3* mutant mice. Furthermore, we determined that both the *FoxO1* and *Smad3* mutant teeth exhibit changes in the expression of similar cohort of genes encoding enamel matrix proteins required for proper enamel development. These data raise the possibility that FoxO1 and Smad3 act in concert to regulate a common repertoire of genes necessary for complete enamel maturation. This study is the first to define an essential role for the FoxO family of transcription factors in tooth development and provides a new molecular entry point which will allow researchers to delineate novel genetic pathways regulating the process of biomineralization which may also have significance for studies of human tooth diseases such as amelogenesis imperfecta.

## Introduction

The process of biomineralization is observed throughout metazoans and results in the generation of biologically important tissues such as shells, carapaces, spicules, bones and teeth [1]. The biomineralization of the mammalian tooth is a particularly striking case as dental enamel contains less than 1% organic matter by weight and is predominantly composed of a highly ordered lattice of calcium hydroxyapatite (Ca_10_(PO_4_)_6_(OH)_2_) crystals making it the hardest mineralized tissue known [2]. Prior to the development of the mature protein-free enamel structure, enamel formation consists of specific cellular events termed the secretory, transition and maturation stages [3], [4]. During the secretory stage, specialized, ectodermally-derived cells called the ameloblasts deposit a complex extracellular matrix composed of amelogenin, ameloblastin, enamelin and other proteins [5], [6], [7]. These enamel matrix proteins are thought to promote the formation and elongation of thin ribbons of hydroxyapatite crystallites which lengthen parallel to one another while not growing in width [8]. At the end of the secretory stage, the enamel ribbons have reached their full-length thus defining the ultimate thickness of the mature enamel layer. Next, during the transition stage, the ameloblasts reduce their deposition of extracellular matrix proteins and convert from the columnar-shaped secretory ameloblasts to the more shortened maturation ameloblasts. It is during the maturation stage that minerals are deposited on the sides of the fully elongated crystallites resulting in an increase in thickness and width. This structure is further elaborated until the thickening parallel crystals come into contact with adjacent crystals [8], [9].

Genetic studies of amelogenesis imperfecta patients and mice with mutations in the genes encoding the enamel matrix proteins show that these proteins are critical for the proper growth and maturation of enamel crystals [10], [11], [12], [13], [14], [15], [16], [17]. However, these proteins are not part of the mature enamel structure. To complete mineralization of the enamel matrix, in which enamel rods grow firmly against one another and become mechanically interlocked, the extracellular matrix components must be digested and reabsorbed by the ameloblasts [8]. In the mouse, both Matrix metalloproteinase 20 (Mmp-20) and Kallikrein 4 (Klk4) have been shown to have enamel matrix protein protease activity *in vivo* and both *Mmp-20* and *Klk4* knockout mice exhibit malformed enamel [18], [19]. Also, mutations in *MMP-20* and *KLK-4* were reported in amelogenesis imperfecta patients [20], [21], [22], [23], [24]. Interestingly, Mmp-20 is expressed and deposited within the extracellular matrix by the ameloblasts during the secretory stage coincident with Amelogenin, Ameloblastin and Enamelin. It is believed that Mmp-20 cleavage of the extracellular matrix permits hydroxyapatite crystal elongation rather than thickening [8]. *Klk4* is expressed beginning at the transition stage and into the maturation stage; however, it has not been detected in secretory ameloblasts [19]. Klk4 is thought to catalyze the proteolytic degradation of the residual extracellular matrix thereby providing additional free space for the elongated enamel crystals to expand in width, contact adjacent crystals and interlock [19]. Overall, the process of enamel biomineralization requires tight spatial and temporal control of numerous genes which also interact post-translationally. Thus, we hypothesized that an ameloblast-specific mechanism of transcriptional regulation likely coordinates this highly specialized developmental process.

Previously, the Smad3 transcriptional co-factor was implicated in the regulation of biomineralization *in vivo*, as mice with a targeted mutation in *Smad3* exhibit a hypomineralized tooth phenotype [25], [26]. Given the well-documented role of Smad3 (together with the co-Smad, Smad4) as an intracellular molecule that mediates signaling from the Transforming growth factor-β (Tgf-β) receptor, we reasoned that Tgf-β signaling might activate a repertoire of genes necessary for the coordination of biomineralization [27]. Consistent with this idea, many studies have already implicated Tgf-β signaling as being important for proper craniofacial development, including tooth formation [28]. However, due to various functional redundancies between Tgf-β signaling molecules, receptors, Smads and Smad transcriptional co-factors, a complete picture of Tgf-β signaling as it relates to specific cellular events during tooth development and maturation remains elusive. Further complicating the issue is the multifunctional role Tgf-β signaling can serve in any given tissue. Thus, targeted deletion of Tgf-β components in mice often results in early embryonic lethality or other pleiotropic effects which make it difficult to assign specific functions to specific components of the pathway.

When considering the broad transcriptional changes which generally occur in response to Tgf-β, how is the Tgf-β signal interpreted by differentiated ameloblasts as a cue to coordinate the expression of specific genes essential for the completion of such specialized developmental processes as enamel biomineralization? Due to studies suggesting that the Smad complex alone is usually insufficient for target gene binding and activation [29], transcription target specificity likely depends on the association with specific Smad transcriptional co-factors expressed coincidentally within the ameloblasts. Members of the forkhead box O family of transcription factors, which serve diverse roles such as cellular growth and proliferation, development, metabolism and longevity [30], have been shown to function as Smad co-factors *in vitro* [31], [32], [33]. Here, via a conditional knockout approach, we show that ameloblast-specific loss of Forkhead box O transcription factor 1 (FoxO1) results in an enamel hypomaturation phenotype that is reminiscent of the *Smad3* mutant [25]. Furthermore, we also show that both *FoxO1* and *Smad3* mutants exhibit a reduction in the expression of a similar cohort of genes encoding enamel matrix proteins. These data raise the possibility that FoxO1 acts as a Smad co-factor, within an ameloblast-expressed transcriptional complex, to regulate a specific set of genes required for proper enamel biomineralization.

## Results

### Ameloblast-specific loss of FoxO1 results in a white tooth phenotype

**Figure 1:**
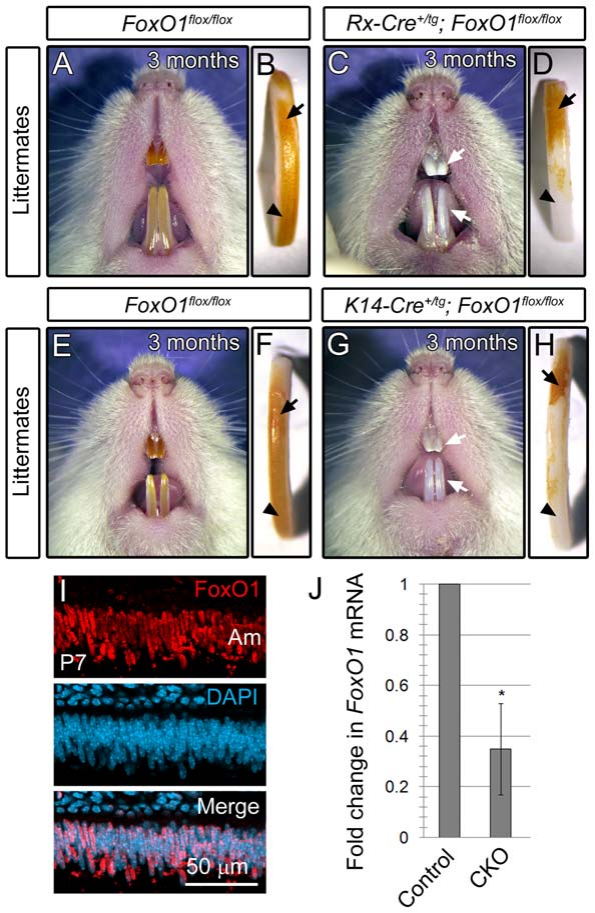
Gross phenotype of the *FoxO1* mutant teeth. Rx-Cre and K14-Cre-mediated knockout of *FoxO1* results in viable, fertile mice exhibiting abnormally white, chalky incisors (compare A to C and E to G). Extracted incisors had intact enamel on the un-erupted surface of the incisor that appeared similar to controls (arrows in B, D, F and H). However, the most distal, erupted region of the mutant incisors was white in appearance suggesting enamel attrition due to occlusal forces (arrowheads in B, D, F and H). FoxO1 is expressed in differentiated postnatal ameloblasts (Am) (I). qrtPCR analysis of postnatal conditional knockout whole incisors confirmed the expected reduction of *FoxO1* transcript (J).

In an independent study, we aimed to address the role of *FoxO1* in retinal development by crossing the *FoxO1* floxed allele [34] to the *Rx-Cre* recombinase line [35]. The *Rx-Cre^+/tg^*; *FoxO ^flox/flox^* mice were born at a normal Mendelian frequency and were viable and fertile. However, the retinae in these mice had normal thickness and the cellular composition was indistinguishable from controls (data not shown). Upon further examination of the mutant mice, we found that 100% of the *FoxO1* conditional knockouts exhibited a chalky, white tooth phenotype that was first apparent by 1 month postnatally and more pronounced by 3 months (Figure 1A, 1C). Extracted maxillary incisors showed that the proximal, immature enamel of the un-erupted mutant incisor appeared yellowish-brown in color similar to controls (Compare Figure 1B to 1D, arrows). However, in the more distal incisor where the tooth has erupted, we noticed that the *FoxO1* mutants were progressively whiter toward the incisal edge (Compare Figure 1B to 1D, arrowhead). This finding suggested that the enamel on *FoxO1* mutant teeth experienced extensive attrition due to chewing. Importantly, we also analyzed two control groups, *Rx-Cre^+/tg^*; *FoxO^+/flox^* and *Rx-Cre^+/tg^* mice and they were found to be phenotypically normal (data not shown).

Given the documented forebrain and retinal-specific activity of Rx-Cre [35], a tooth phenotype was surprising. Since the *Rx-Cre* is expressed within the forebrain early in development, it is possible that Cre expression is broader than previously appreciated, encompassing precursors of the enamel producing ameleoblasts. To show that *FoxO1* is required in ameloblasts, we crossed the *FoxO1* floxed allele to mice carrying the *Keratin14 (K14)-Cre* transgene, which has been shown to drive Cre activity within ectodermally-derived tissues including the ameloblasts [36], [37]. As expected, *K14-Cre^+/tg^*; *FoxO1^flox/flox^* mice also exhibited a white tooth phenotype similar to the *Rx-Cre^+/tg^*; *FoxO1^flox/flox^* mutants (Compare Figure 1E to 1G, arrows). These mutants also showed the same progressive proximal to distal whitening of the incisor surface (Compare Figure 1F to 1H).

To further confirm a role for *FoxO1* in the mouse ameloblasts, we labeled cryosections of postnatal day 7 (P7) incisors with anti-FoxO1 antibodies and 4′,6-diamidino-2-phenylindole (DAPI) nuclear dye. Upon confocal microscopy, we found that FoxO1 was expressed ubiquitously throughout the mouse incisors and molars and co-localized with the DAPI signal (Figure 1I and data not shown). The nuclear expression of FoxO1 was consistent with the known role of FoxO1 as a transcription factor. Furthermore, we performed quantitative reverse transcriptase polymerase chain reaction (qrtPCR) on mRNA isolated from adult *FoxO1* conditional mutant and control incisor enamel organs and found that the *FoxO1* conditional mutants exhibited a 65% reduction (P<0.001) in *FoxO1* mRNA levels relative to the controls (Figure 1J). Given the broad expression of *FoxO1*, the residual *FoxO1* mRNA observed in the conditional mutants is likely due to the presence of cells outside the ameloblast layer which did not undergo Cre-mediated recombination.

**Figure 2:**
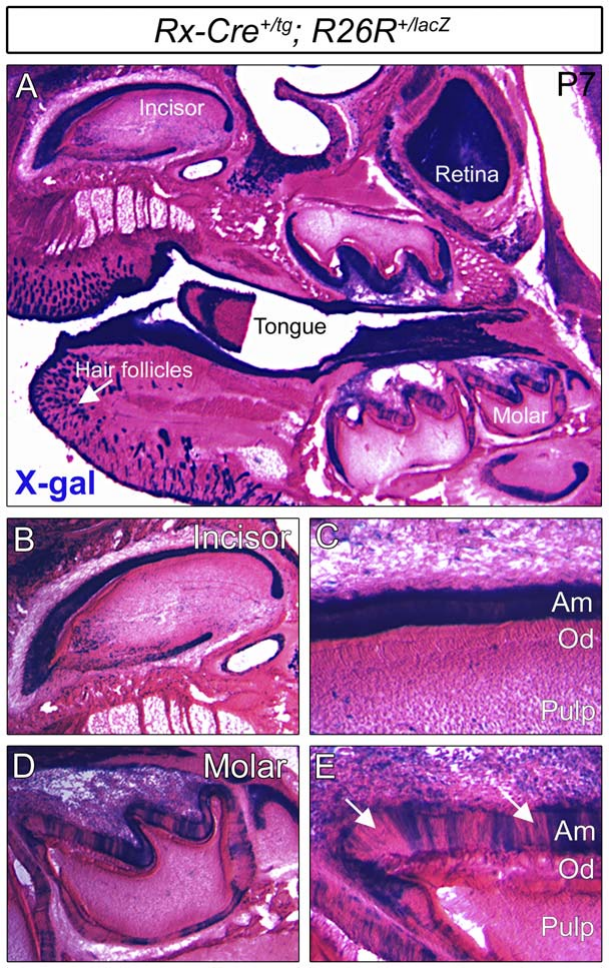
Within the tooth, Rx-Cre activity is confined to the ameloblast layer. *Rx-Cre^+/tg^* mice were crossed to the *ROSA26^+/lacZ^* Cre reporter line and Cre activity was assessed via X-gal staining. Rx-Cre exhibited broad activity throughout the anterior head in such tissues as the retina, tongue, skin, hair follicles, and teeth (A). Closer inspection of the incisors (B and C) and the molars (D and E) revealed that Cre activity was confined to the ameloblast layer (Am) and completely absent from the odontoblast layer (Od). The Cre activity in the molars was more mosaic than the incisors (arrows in E).

For final confirmation that the *Rx-Cre^+/tg^*; *FoxO1^flox/flox^* mutant phenotype was due to specific loss of *FoxO1* within the ameloblasts, we crossed the *Rx-Cre* line to mice expressing the ROSA26^+/lacZ^ Cre reporter [38], thus generating *Rx-Cre^+/tg^*; *ROSA26R^+/lacZ^* progeny. Postnatal day 7 heads were cryosectioned and ROSA26^+/lacZ^ Cre reporter activity was assessed via 5-bromo-4-chloro-3-indolyl-β-D-galactopyranoside (X-gal) staining. As expected, we observed lacZ activity in a wide array of tissues within the head and facial regions including hair follicles, retina, skin, oral epithelium, tongue, bone and teeth (Figure 2A and data not shown). Within the teeth, lacZ expression was confined predominantly to the ameloblast layer and we did not observe expression within the odontoblasts (Figure 2B–C). Furthermore, the ameloblasts of the incisors exhibited uniform expression of lacZ whereas the molars had a variable mosaic pattern of expression in which regions of the ameloblast layer were negative for lacZ (Compare Figure 2C to 2E, arrows). The basis for the mosaic Cre activity in the molars is not known.

### FoxO1 mutant tooth enamel is softer than controls

**Figure 3:**
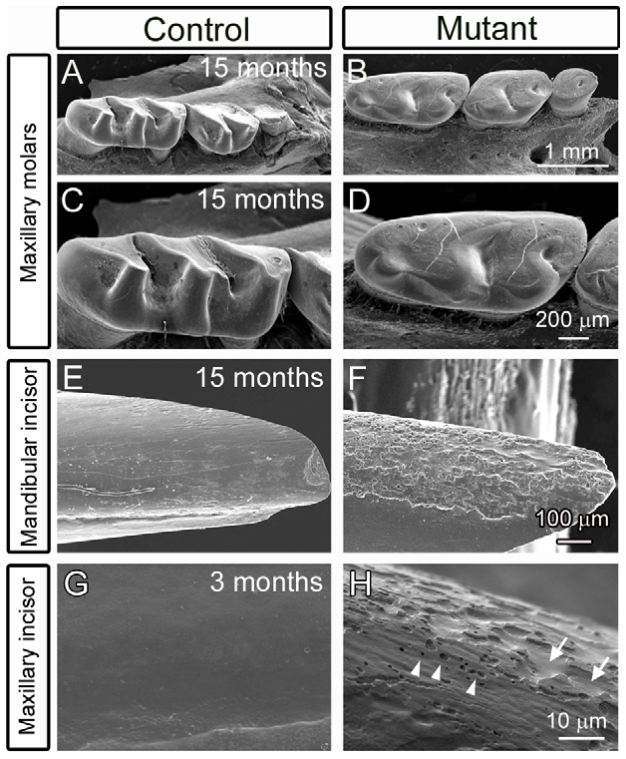
*FoxO1* mutant teeth suffer from severe enamel attrition. Scanning electron microscopy revealed that 15 month old *FoxO1* mutant molars displayed a pronounced wearing of the enamel layer to the extent that the molar cusps had almost completely eroded away (compare A and B and high magnification views in C and D). The 15 month old mutant incisors had a similar phenotype in which the labial surfaces of both the maxillary (shown) and mandibular (not shown) incisors had a dramatic chipping of the enamel from the dentin layer giving the incisors a rough appearance (compare E to F). This phenotype was also seen as early as 3 months (compare G to H) and higher magnification revealed discrete valleys (arrows in H) and holes (arrowheads in H) in the enamel surface.

**Figure 4:**
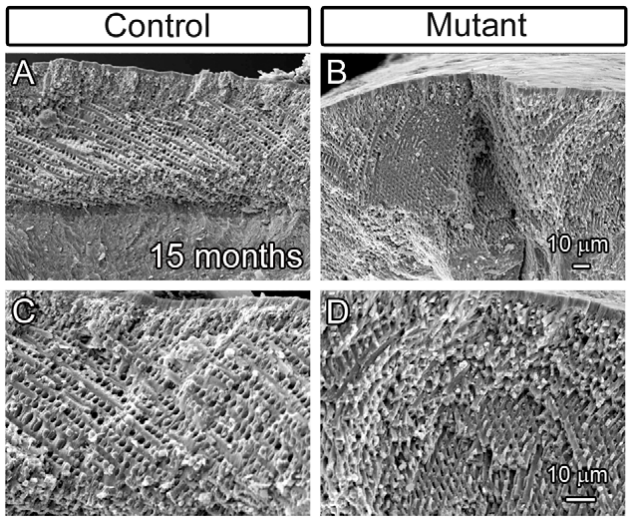
*FoxO1* mutant teeth deposit an enamel matrix and form enamel rods. Scanning electron microscopy of fractured 15 month old *FoxO1* mutant incisors revealed that, in regions where enamel had not chipped away, the mutant teeth initially had an enamel thickness that was comparable to their littermate controls (compare A and B). Higher magnification revealed that the mutant enamel also exhibited the typical decussating pattern of enamel rods (compare C and D).

Given the white tooth phenotype of the *FoxO1* mutants and activity of *Rx-Cre* and *K14-Cre* within the ameloblasts, we reasoned that *FoxO1* deletion within the ameloblasts led to hypomaturation of the enamel layer. In order to examine the enamel structure in greater detail, control and *FoxO1* mutant maxillae and mandibles were removed and the periradicular bone was dissected away. The exposed molars and incisors were analyzed via scanning electron microscopy (SEM). At 15 months, the *FoxO1* mutant molars exhibited dramatic attrition of the enamel surface such that the cusps of the molars were smooth in appearance as compared to controls (Figure 3A–D). We also examined the surface of the mandibular incisors and found that the *FoxO1* mutants had a rough appearance in stark contrast to the smooth surface of the control incisors (Compare Figure 3E to 3F). Analysis of maxillary incisors of 3 month old mutants also revealed a similar defect (Figure 3G and 3H). Upon close inspection, regions of the enamel surface appeared to erode away thereby creating the appearance of valleys (Figure 3H, arrows). We also observed small, discrete holes within the enamel surface ranging from approximately 0.5 to 2.0 microns in diameter (Figure 3H, arrowheads). Taken together, these data suggest that the *FoxO1* mutant tooth enamel is weaker than the control mice and erodes away over time. However, subsequent imaging of mutant enamel layer cross-sections in fractured incisor preparations revealed that the thickness of the enamel layer as well as the decussating pattern of the enamel rods appeared similar to the controls (Figure 4A–D). This finding suggested that the secretory stage ameloblasts of the *FoxO1* mutants are at least partially functional. However, despite substantial enamel matrix deposition, our data indicated that the *FoxO1* mutant enamel does not undergo normal maturation resulting in compromised enamel strength.

**Figure 5:**
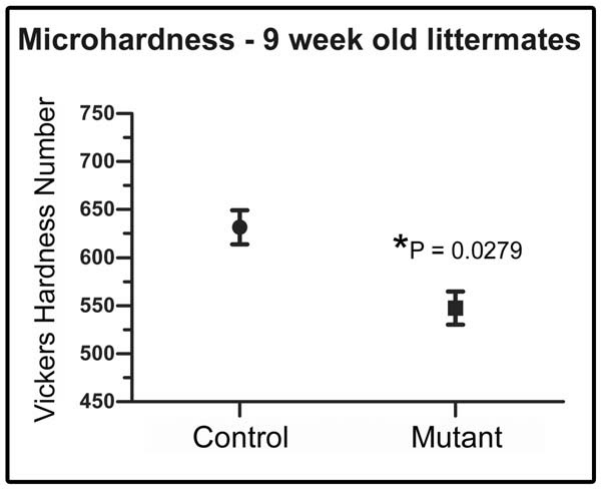
*FoxO1* mutant enamel is softer than controls. The Vickers microhardness test revealed that the adult *FoxO1* mutant teeth are significantly softer than their littermate controls.

In order to confirm our interpretation of the *FoxO1* mutant SEM data as indicating an enamel hypomaturation phenotype, we next attempted to determine whether the *FoxO1* mutant enamel is softer than controls. To do this, we subjected littermate control and mutant teeth to an enamel microhardness test. Micro-indentations were created on maxillary incisors from 9 week old *FoxO1* mutant and control mice (n = 3 per group). The average hardness for control enamel was found to be 631.4 VHN (SEM ± 17.78) whereas the *FoxO1* mutant enamel was 547.7 VHN (SEM ± 17.28). The difference was statistically significant (p = 0.0279) and the results demonstrated that the mature mutant enamel was approximately 13.3% softer than control enamel (Figure 5). We attempted the same analysis on 15 month old teeth, but we were unable to identify large enough, intact enamel surfaces on which to perform the test.

### FoxO1 loss-of-function does not affect gross ameloblast development or differentiation

**Figure 6:**
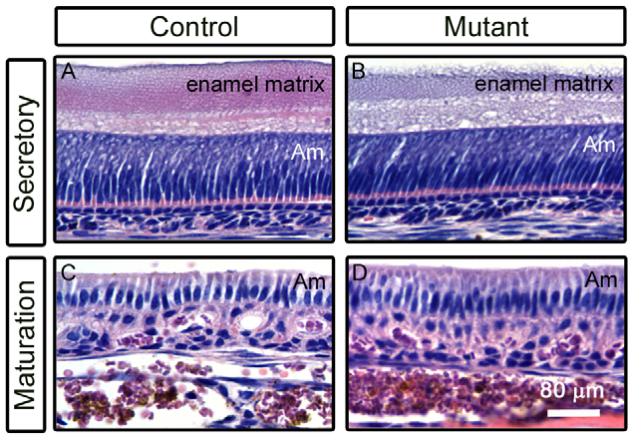
The morphology of *FoxO1* mutant ameloblasts appears unaffected. Hematoxylin and EosinY staining of paraffin sections of decalcified control and *FoxO1* adult mutant teeth showed that mutant secretory stage (A and B) and maturation stage (C and D) ameloblasts (Am) appeared structurally similar to controls.

**Figure 7:**
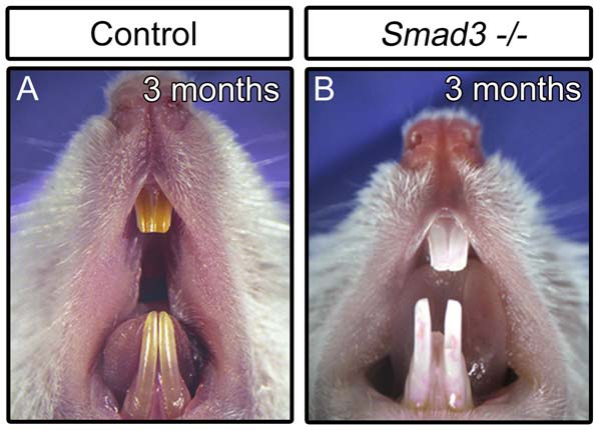
*Smad3* mutants suffer from a white, chalky tooth phenotype reminiscent of the *FoxO1* mutants.

Since our data support a requirement for *FoxO1* in ameloblasts, we next sought to determine whether the *FoxO1* mutants exhibited a decrease in ameloblast density, failure of polarization or a failure to transition between different stages of amelogenesis that could explain the resulting weakened enamel structure. To assess ameloblast cytoarchitecture, we dissected and decalcified maxillary and mandibular incisors from 15 month old control and *FoxO1* mutant littermates and performed hematoxylin and eosin-Y (H&E) staining on paraffin sections. Interestingly, in control and mutant mice, both the secretory and maturation stage ameloblast layers appeared very similar in terms of overall cell density, organization and polarization (Figure 6 A–D). Thus, it is unlikely that the observed *FoxO1* mutant soft enamel phenotype is due to defects in ameloblast genesis and/or differentiation. Interestingly, *Smad3* mutants show a very similar white tooth phenotype yet also fail to exhibit obvious defects in the morphology of the ameloblast layer (Figure 7) [25].

### FoxO1 and Smad3 mutants exhibit dysregulation of a similar cohort of genes necessary for biomineralization

**Figure 8:**
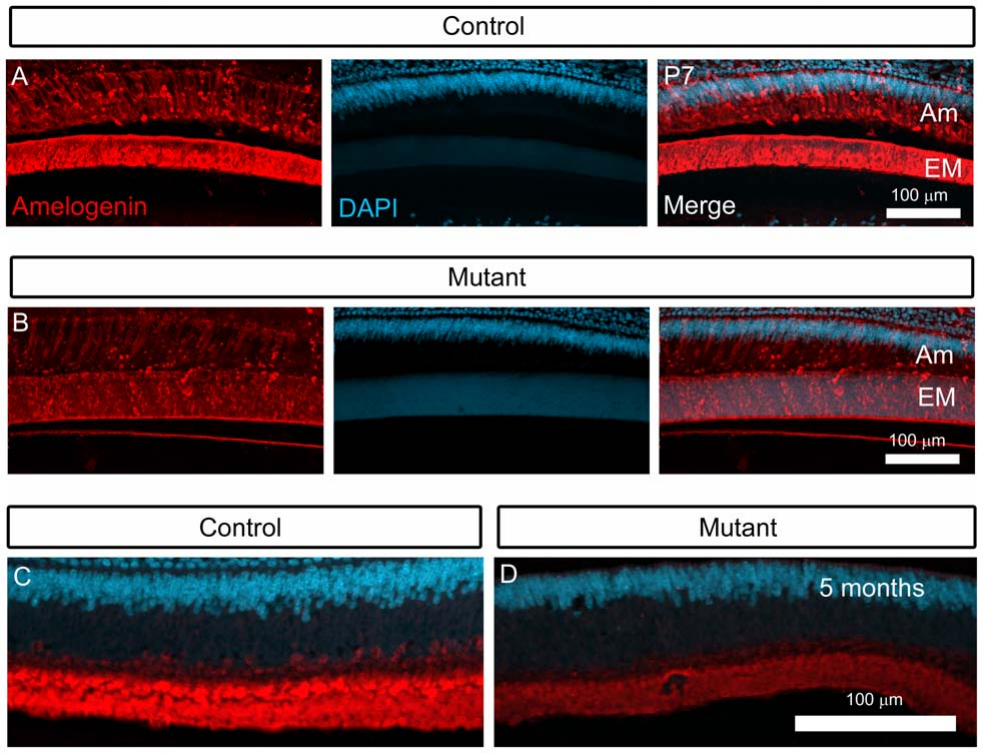
*FoxO1* mutant incisors exhibit an apparent reduction in amelogenin protein expression. Immunofluorescent analysis of cryosections derived from P7 and 5 month old *FoxO1* and littermate control mice revealed that *FoxO1* mutants exhibit a qualitative reduction in Amelogenin protein expression. This reduction in fluorescent signal was observed in both the ameloblasts (Am) and the enamel matrix (EM).

**Figure 9:**
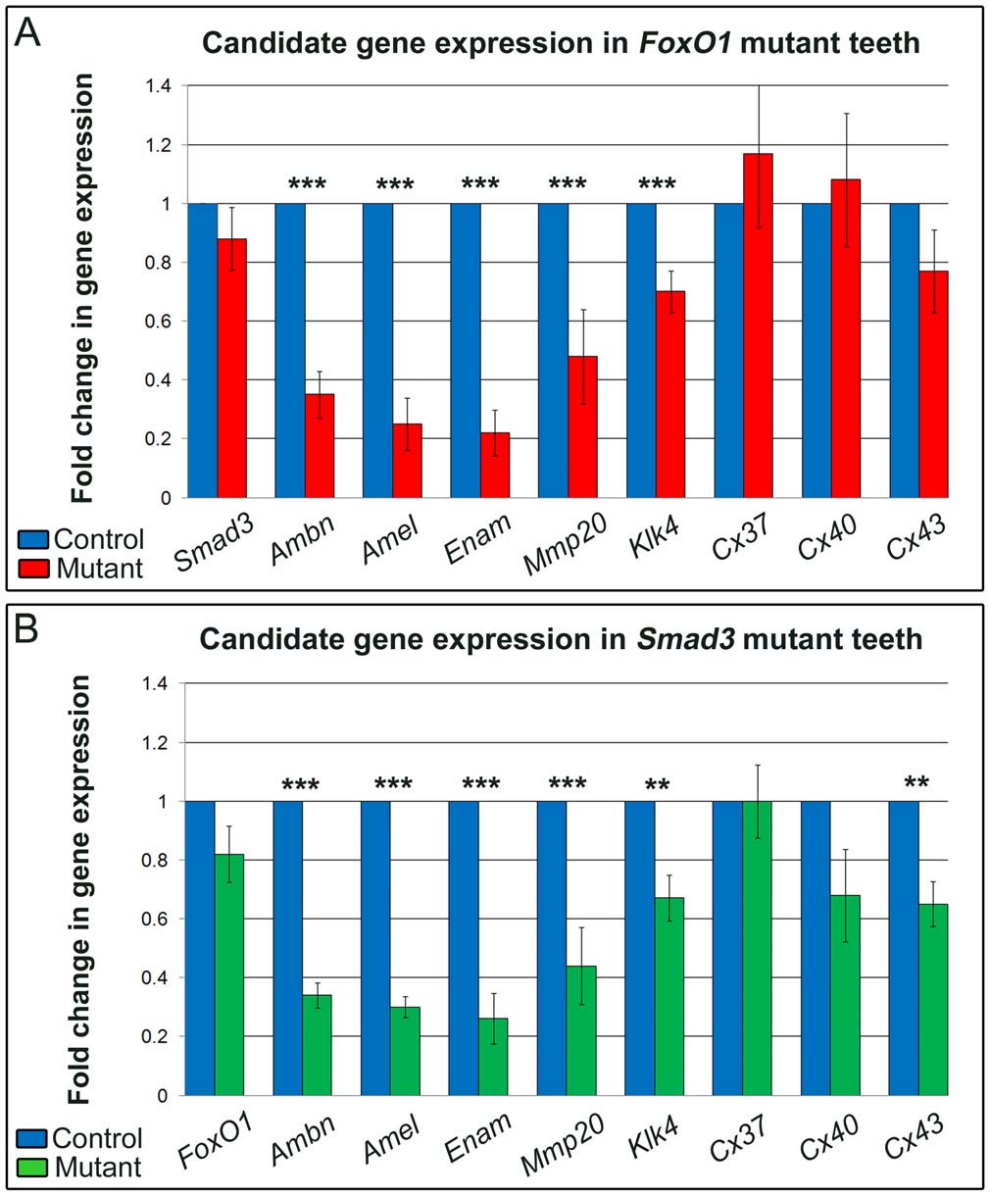
Loss of *FoxO1* and *Smad3* results in the down-regulation of a common set of genes. Quantitative rtPCR was performed on mRNA isolated from adult *FoxO1* mutant (A) and *Smad3* mutant (B) incisors and compared to control littermates. Both mutants exhibited a similar trend in the down-regulation of genes known to be necessary for proper enamel development and maturation (*p<0.05, **p<0.01, ***p<0.001).

When considering the known role of *FoxO1* as a transcription factor, we next sought to address whether FoxO1 regulates a set of genes that might provide insight into the enamel hypomaturation phenotype. Since the *FoxO1* mutants failed to exhibit an obvious change in ameloblast specification and differentiation, we decided to focus our analysis predominantly on genes that have been directly implicated in the process of enamel biomineralization. Since Amelogenin proteins constitute 90% of the ameloblast extracellular matrix and are essential for normal enamel organization and maturation [11], [39], [40], we began our analysis by assessing Amelogenin protein expression in *FoxO1* mutant and control teeth. Using antibodies against Amelogenin, we performed immunofluorescent analysis of cryosections from 7 day and 5 month old *FoxO1* mutant and control incisors. Confocal microscopy suggested that the *FoxO1* mutant Amelogenin protein expression levels were reduced as compared to littermate controls (compare Figure 8A to 8B and Figure 8C to 8D). While immunofluorescence provides spatial information regarding gene product expression, it cannot be reliably used as quantitative assessment. Thus, we next performed qrtPCR analysis of adult *FoxO1* mutant and control incisors. Given the previously documented role of FoxO transcription factors as Smad transcriptional co-activators [31], [32], [33], the expression of FoxO1 and Smads within endogenous ameloblasts [41], [42], [43] and the white tooth phenotype of the *FoxO1* and *Smad3* mutant mice (Figure 1 and Figure 7) [25], we decided to include *Smad3* mutant teeth in our qrtPCR analysis. We extracted adult maxillary and mandibular incisors from *FoxO1* mutant, *Smad3* mutant and control mice and purified mRNA for subsequent qrtPCR analysis. It should be noted that littermate controls were used in every case so that genetic background was consistent with that of the respective mutant. Upon analysis of transcript levels, we found that *Ameloblastin (Ambn)*, *Amelogenin (Amel)*, *Enamelin (Enam)*, *Mmp20* and *Klk4* mRNA levels were all significantly reduced in both the *FoxO1* and *Smad3* mutant mice as compared to controls with the enamel matrix proteins (including amelogenin) showing the greatest reduction (Figure 9A–B). Furthermore, the relative reduction of *Ameloblastin*, *Amelogenin*, *Enamelin*, *Mmp20* and *Klk4* expression had a very similar trend in both *FoxO1* and *Smad3* mutants with *Enamelin* being the most reduced and *Klk4* being the least reduced. Since *FoxO1* loss-of-function in other tissues during development has been shown to cause a dramatic reduction in the expression of *Connexin-37 (Cx37)* and *Connexin-40 (Cx40)* mRNA levels (47), we included these genes in our analysis [44]. We also included *Connexin-43 (Cx43)* as *Cx43* mutants exhibit an enamel maturation defect [45], [46], [47]. Loss of *FoxO1* or *Smad3* has no effect on the levels of *Cx37* or *Cx40* mRNA (Figure 9A–B). The level of *Cx43* was modestly down-regulated in the *Smad3* mutants but not significantly reduced in the *FoxO1* mutants. Finally, we determined that *Smad3* is normally expressed in the *FoxO1* mutants and *FoxO1* is normally expressed in the *Smad3* mutants. Taken together, these data suggest that FoxO1 and Smad3 potentially regulate a common set of genes necessary for enamel formation and maturation.

### In silico analysis of the enamel matrix proteins revealed conserved FoxO and Smad binding elements

**Figure 10:**
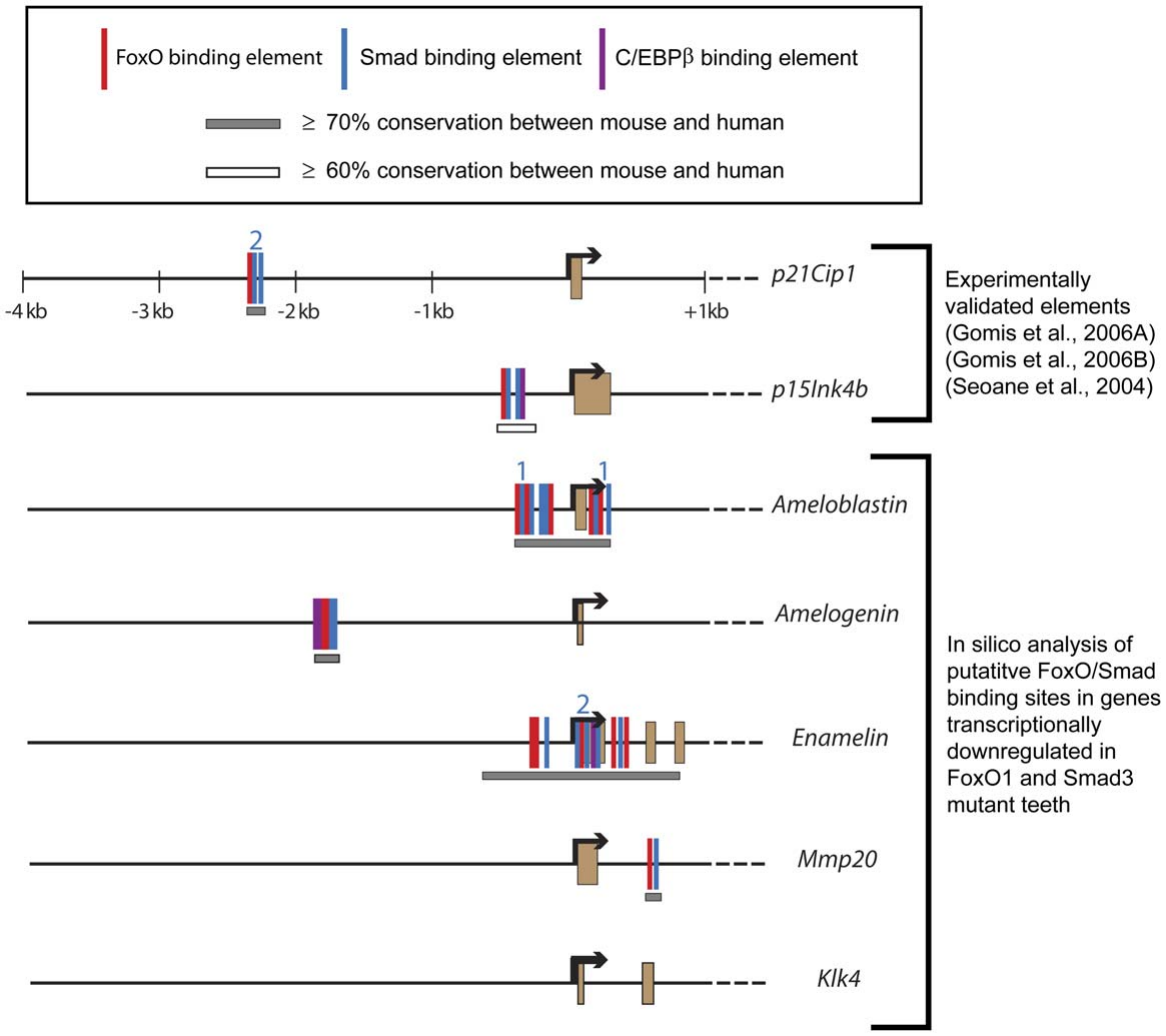
Mapping of putative FoxO/Smad genomic binding sites. *In silico* analysis of putative FoxO, Smad, and CEBPβ binding elements, conserved between mice and humans and residing within several genes down-regulated in the *FoxO1* and *Smad* mutants (see text for further details). The colored numbers indicate additional SBEs or CBEs that, due to scaling, could not be represented on the gene tracks.

Next, using a method similar to previous studies [31], [32], [33], we performed *in silico* analysis to map putative FoxO and Smad binding elements within the genes that showed a transcriptional decrease in the *FoxO1* and *Smad3* mutants (Figure 10). As a positive control, to ensure our search method was comparable to previous methods, we included searches of regions upstream of *p21Cip1* and *p15Ink4a* as these have been previously mapped and were validated experimentally [31], [32], [33]. Also, since its binding sites are occasionally located within FoxO/Smad binding site clusters [32], [33], we searched for elements recognized by the transcription factor C/EBPβ (CBEs). For this analysis, we used mouse genomic sequence from 4 kb upstream of the transcription start site to the stop codon of each gene. FoxO binding elements (also know as forkhead-binding elements or FHBEs) [(G/A)(T/C)AAA(T/A)A] and Smad binding elements (SBEs) [AGAC] (within 100 nucleotides upstream and downstream of the FoxO elements) were then identified using the dual site matching program. Putative FoxO and Smad elements were localize upstream of the transcription start site of *Ameloblastin*, *Amelogenin* and *Enamelin*. Furthermore, *Amelogenin* contained a single C/EBPβ element within the same FoxO/Smad cluster. Ameloblastin and Enamelin contained additional sites within the first exon and intron. *Mmp20* contained only a single FoxO/Smad cluster and it was localized to the first intron. *Klk4* did not contain any putative FoxO, Smad or C/EBPβ elements. These data suggest that at least a subset of the genes down-regulated in the *FoxO1* and *Smad* mutants may be direct targets of the FoxO/Smad transcriptional complex that has been previously identified in epithelial cells [31], [32], [33] and that, in some cases, C/EBPβ may provide additional regulation.

## Discussion

Here, we have uncovered a novel role for *FoxO1* in enamel maturation as well as a molecular entry point from which to further elucidate the mechanism of transcriptional control over mammalian biomineralization. While the *FoxO1* mutants exhibit soft tooth enamel which undergoes severe attrition over time, we did not observe any obvious dysplasia of the ameloblasts. This phenotype is strikingly similar to that of the *Smad3* mutant mice [25]. Based on such a close phenocopy between these two mutants, we hypothesized that these genes play similar roles in enamel development. Indeed, we demonstrated that the *FoxO1* and *Smad3* mutants exhibit down-regulation of a similar cohort of genes known to be important for enamel development and maturation. Previously, in *in vitro* studies of epithelial cells, it was shown that FoxO and Smad proteins exist in a TGF-β-dependent transcriptional complex which regulates the expression of a common set of genes termed a synexpresison group [31], [32], [33]. These genes comprise a functionally diverse group including mediators of cellular stress and cystostatic responses. This finding raises the possibility that a similar transcription factor complex functions in ameloblasts (the epithelial component of the tooth) to regulate enamel matrix protein expression. Consistent with this idea, *in silico* mapping of FoxO and Smad binding sites uncovered clusters of conserved regulatory elements located upstream of the transcriptional start sites of *Ameloblastin*, *Amelogenin* and *Enamelin*. Interestingly, in the *FoxO1* and *Smad* mutants, these three genes also showed a greater reduction in expression levels as compared to *Mmp20* and *Klk4* proteases which did not contain upstream FoxO or Smad binding elements. These data raise the possibility that a FoxO1/Smad3 complex may directly regulate a subset of genes that must be expressed in an overlapping temporal and spatial manner and serve a similar role during enamel development. However, unequivocal demonstration of such a complex directly regulating the expression of enamel matrix proteins in ameloblasts *in vivo* awaits FoxO1/Smad3 co-immunoprecipitation and chromatin immnoprecipitation analyses of adult mouse incisors.

Surprisingly, loss-of-function studies of genes encoding the enamel matrix proteins (Ameloblastin, Amelogenin, and Enamelin) exhibit a phenotype which is more severe than the *FoxO1* or *Smad3* mutants. These mutants have dramatic defects in the development of the enamel in which the normal prismatic enamel structure is severely disrupted or the enamel completely fails to form [10], [11], [14], [48], [49]. In the *FoxO1* mutant teeth, the enamel prisms form but fail to become fully mineralized resulting in a weakened structure. Furthermore, while the *Ameloblastin*, *Amelogenin*, and *Enamelin* mutant ameloblasts show morphological abnormalities such as loss of cell polarity, cyst-like structures and detachment from the extracellular matrix [10], [11], [14], [48], [49], the *FoxO1* and *Smad3* ameloblasts appear morphologically normal [25]. The relatively mild defects observed in the *FoxO1* and *Smad3* mutants are likely explained by the finding that these mutants still express appreciable levels of the major enamel matrix proteins. However, the levels are significantly reduced from wild type. It is worth noting that the *Enamelin* knockout mice exhibit an autosomal dominant enamel phenotype that appears less severe than the *FoxO1* and *Smad3* mutants [13]. The mandibular incisors of these mice were white and chalky in appearance and exhibited attrition of the erupted portions. However, as in the *FoxO1* mutant mice, the enamel prisms looked normal. These data suggests that the *Enamelin* heterozygous mutant enamel forms but is softer than normal. Interestingly, among all the putative *FoxO/Smad* target genes analyzed in this study, *Enamelin* mRNA levels showed the greatest reduction. Thus, it is formally possible that the reduction of *Enamelin* expression is the main contributing factor to the *FoxO1* and *Smad3* mutant enamel phenotypes.

In a recent study, FoxO1 was shown to directly induce the expression of transcription factor *Runx2 (Cbfa1)* as well as physically associate with Runx2 protein to regulate genes essential for osteoblast differentiation and skeletogenesis [50]. Of further significance is the finding that Runx2 has the ability to bind to regions of the *Ameloblastin* promoter and possibly participate in the control of *Ameloblastin* transcription [51]. Also, *Runx2* was shown to be endogenously expressed in secretory and maturation ameloblasts, and loss of *Runx2* expression results in severely hypoplastic teeth lacking definitive odontoblast and ameloblast differentiation [52], [53], [54], [55]. Thus, it is possible that FoxO1 and Runx2 may be functionally related during enamel maturation. However, we performed qrtPCR to assess the levels of *Runx2* expression in *FoxO1* mutant incisors and found that *Runx2* (and *Runx1*) levels were the same as controls (not shown). It is also important to recognize that the documented interaction of FoxO1 and Runx2 during osteoblast differentiation occurs in mesenchymal cells [50] while enamel formation is driven by the ameloblasts, which are epithelial cells. Thus, one can envision dramatic differences in terms of Runx2 function depending on the specific cellular context. Indeed, it has been shown that TGF-β signaling to osteoblasts, via Smad3, has the ability to repress the transcription of *Runx2* as well as Runx2 protein activity [56], [57]. However, whether this particular function of Smad3 is FoxO-dependent was not addressed. Other transcription factors potentially functioning in concert with FoxOs or Smads in enamel maturation are C/EBPβ and related family members. One member, C/EBPα, was shown to be a transcriptional activator of the mouse *Amelogenin* gene *in vitro*. Interestingly, ameloblast specific ablation of *C/EBPα* did not result in an enamel phenotype or loss of *Amelogenin* expression and this was shown to be due to redundancy with *C/EBPδ* [58], [59]. By employing future bioinformatic experiments to map FoxO/Smad binding elements, we should be able to uncover more putative target genes. By combining this approach with subsequent conditional knockout of target genes and functional validation of direct binding sites, we will gain increasingly better insight into the process of enamel formation and maturation *in vivo*.

Our data show that *FoxO1* mutant ameloblasts are morphologically normal yet the enamel is weaker than controls. This suggests to us that *FoxO1* likely does not function in ameloblast development, but rather in the production of mature enamel. While it is formally possible that low or mosaic Cre activity at early tooth developmental stages might preclude the discovery of a subtle, earlier function of *FoxO1* in ameloblast specification and differentiation, we do not believe this scenario to be likely. The *K14-Cre* transgene has been shown by several groups to be active at the E12 tooth bud stage which is several days before ameloblast differentiation occurs [60], [61] and K14-Cre-mediated conditional knockouts of other genes have been shown to disrupt ameloblast differentiation and organization [61], [62]. Furthermore, it is well-known that rodent incisors contain a stem cell niche called the cervical loop from which ameloblasts continuously renew throughout the lifetime of the animal [63], [64]. It is also known that K14-Cre and Rx-Cre (shown in our study, Fig. 2B) are active in the cervical loop [36]. Thus, the ameloblast stem cell population would be expected to lose *FoxO1* expression thereby giving rise to *FoxO1*-deficient ameloblasts in the adult. Since, we only observed an enamel maturation defect in the mutant mice, this would argue against a role for *FoxO1* in ameloblast differentiation.

Our study of the role of *FoxO1* in mouse ameloblasts has established a critical molecular entry point which will allow researchers to delineate novel genetic pathways regulating the process of biomineralization. FoxO1 and Smads, either directly or indirectly, likely control the expression of numerous genes, possibly in addition to enamel matrix proteins, which are essential for the completion of tooth mineralization. Some of these genes may also be dysregulated in human diseases such as amelogenesis imperfecta.

## Materials and Methods

### Mouse Strains

*FoxO1^flox/flox^* [34], *Rx-Cre ^flox/flox^* [35], *K14-Cre ^flox/flox^* [65], and *ROSA26R^+/lacZ^* [38] mice were maintained on a mixed C57BL/6J, FVB/NJ background. *Smad3−/−* mice were maintained on a BALB/c background [26]. PCR genotyping was performed as described in the references indicated above. All animal research was conducted according to protocols approved by the Institutional Animal Care and Use Committee (IACUC) of Baylor College of Medicine (assurance number 3823-01).

### Scanning Electron Microscopy

Mandibular and maxillary incisors from 3 animals at each stage and genotype were mounted whole or were fractured and then mounted on aluminum stubs to expose the fractured plane. Samples were sputter-coated for analyzing enamel structure on a scanning electron microscope (JEOL).

### Microhardness Test

Erupted portions of maxillary incisors from 3 *FoxO1* mutant and 3 control littermates were fixed overnight in 100 mg/ml formaldehyde, 10 g/L zinc sulfate, washed and dehydrated with grade alcohol and acetone. Incisors were embedded sagittally in hard-formulation epoxy embedding medium (EpoFix, EMS). Samples were ground and polished to 0.25 µm with a diamond suspension (EMS). The polished samples were tested for enamel microhardness on a Leco M 400 HI testing machine (Leco). Testing was performed with a load of 25 g for 5 sec with a Vickers tip. Twenty-five indents per sample were measured for hardness. Statistical significance was determined by one-way student's t-test (GraphPad Prism 5).

### RNA extraction and quantitative rtPCR

Adult control and mutant incisors (n = 3 per genotype), removed from the surrounding bone and with the enamel organ intact, were homogenized in liquid nitrogen using a mortar and pestle. The ground samples were further homogenized in Trizol (Invitrogen) and total RNA was extracted according to the manufacturer's protocol. RNA was subsequently DNAse (Invitrogen) digested to eliminate genomic DNA, and purified using the RNeasy RNA clean up kit (Qiagen). The purified RNA was reverse transcribed using the Superscript III first strand synthesis kit with Oligo(dT)_20_ and random hexamer priming (Invitrogen). The Taqman® gene expression assay from Applied Biosystems was used for qrtPCR, and primer pairs from Applied Biosystems were used to detect gene expression: *Gja1/Cx43* (Mm00439105_m1), *Gja5/Cx40* (Mm01265686_m1), *Gja4/Cx37* (Mm01179783_m1), *Mmp20* (Mm00600244_m1), *FoxO1* (Mm00490671_m1), *Klk4* (Mm00517338_m1), *Amelogenin* (Mm00711644_g1), *Ameloblastin* (Mm00477485_m1), *Enamelin* (Mm00516922_m1), and *Smad3* (Mm00489638_m1). qrtPCR was performed on an Applied Biosystems' ABI PRISM 7000 Real time PCR System under the following PCR conditions: 50°C for 2 min., 95°C for 2 min., 40 cycles of 95°C for 15 seconds and 60°C for 1 minute. To determine relative quantification of gene expression, qrtPCR was performed at different dilutions (0.1 ng–10 ng) in quintuplicate between control and mutant cDNA samples. The data was normalized to a housekeeping gene, *Gapdh* (Applied Biosystems Cat #4352932E). For data analysis, the Pfaffl method was used to determine relative gene expression ratios (Pfaffl MW, 2001).

### H&E staining

Maxillary and mandibular incisors from control and *FoxO1* mutant littermate mice were fixed overnight in Zinc-Formalin, decalcified in 5% formic acid, dehydrated and embedded in paraffin. For staining, sections were re-hydrated and stained with Hematoxylin and EosinY.

### X-gal staining and immunofluorescence

Whole postnatal day 7 mouse heads were fixed in 4% paraformaldehyde for 3 hours at 4°C. After fixation, the heads were washed in 1× phosphate-buffered saline (PBS, pH 7.3) 3 times for 10 minutes at 4°C. Next, the samples were cryoprotected by immersing in 15% and then 30% sucrose until the tissue sank to the bottom of the tubes. Subsequently, the tissue was immersed in a 1∶1 solution of 30% sucrose and OCT medium and left at 4°C for a couple of hours. Then, the tissue was embedded in OCT on dry ice and stored at −80°C prior to sectioning. Cryosections were cut at 20 µm on a cryostat and mounted on Superfrost Plus slides (VWR Brand, Westchester, PA). X-gal staining was performed at 37°C for 6 hours as previously described [66]. For immunofluorescence of cryosections, slides were post-fixed in 4% PFA for 10 minutes and then washed in 1× PBS-T (PBS+0.1% TritonX-100) 3 times for 10 minutes at room temperature. Next, the slides were blocked in 2% normal donkey serum diluted in 1× PBS for 1 hour at room temperature. anti-FoxO1 (sc49437, Santa Cruz) or anti-Amelogenin (sc32892, Santa Cruz) primary antibodies were diluted (1∶200) in the same blocking solution and incubated on the slides overnight at 4°C in a humid chamber. Next, the slides were washed 4 times at room temperature in 1× PBS. Labeling with donkey, anti-rabbit Alexa Fluor 488 secondary antibodies (1∶400) (Molecular Probes) was performed using the same blocking solution (2% donkey serum in 1× PBS) and slides were incubated for 1 hour at room temperature. Slides were then stained with DAPI (1∶500) and mounted with FluoroMount-G (Southern Biotech).

### In silico analysis of FoxO/Smad binding sites

Mouse and human genomic sequences were obtained from the University of California Santa Cruz genome browser (http://genome.ucsc.edu). Genomic sequence from 4 kb upstream of the transcription start site to the stop codon of each gene were extracted from either mouse genome release mm9 (NCBI Build 37, July 2007) or human genome release hg19 (NCBI Build 37, Feb. 2009). Forkhead binding elements (FHBEs) [(G/A)(T/C)AAA(T/A)A] and Smad binding elements (SBEs) (AGAC) within 100 nucleotides upstream and downstream of the FHBE(s) were then identified in the mouse sequences using the dual site matching program (http://cbio.mskcc.org/cgi-bin/lash/dualsite). Binding elements were identified in the dual site program after the following steps were taken: i) The “Invert Sites?” option marked as “yes;” ii) the known 5′-to-3′ sequence of the binding element and its reverse were added into the Primary of Secondary Sites fields (therefore, noting the SBE sequence to be AGAC, one would enter this sequence plus the sequence CAGA into the Secondary Sites field). The identified FHBE-SBE sites detected in the mouse sequences were BLAST-compared against the homologous human gene sequences using NCBI bl2seq (http://blast.ncbi.nlm.nih.gov/Blast.cgi) to confirm conservation between the two species. Conservation of a FHBE-SBE(s) was only noted on Figure 9 if i) the FHBE was completely conserved or the conservation was incomplete by a single nucleotide, ii) if the FHBE conserved between the two species was not identical by nucleotide character but exactly matched published FHBE sequences, iii) if the SBE was completely conserved or the conservation was incomplete by a single nucleotide. The criteria were formulated after determining the nature of published and functionally validated FHBE-SBEs [31], [32], [33]. CBEs [T(G/T)NNG(A/C)AA(G/T)] and SBEs within 100 nucleotides upstream and downstream of the CBE were then identified in a similar manner. CBEs that were located within previously identified FHBE-SBE clusters were singled out and the sequence identity between mouse and human was again detected using the NCBI bl2seq program. As for the FHBE and SBE sequences, CBE sequences were only noted if i) the conservation between mouse and human sequences was complete, ii) incomplete by a single nucleotide, or iii) if the CBE conserved between the two species was not identical by nucleotide character but exactly matched published CBE sequences [32], [33]. Regions of ≥60% or 70% sequence identity larger than the regions encompassing the FHBE-SBE(s) were determined by comparing the entire promoter region of each mouse gene to the human gene using the NCBI bl2seq program. The sequence identity recorded for the *Mmp20* and *Cx40* genes come from the dual site program rather than the NCBI bl2seq program. Sequences for *p21CIP1* and *p15INK4b* were extracted from releases mm6 and hg17, and mm8 and hg17, respectively, to mimic previously published results. It should be noted that the binding sites represented for *p15INK4b* were acquired from published results and were not obtained using the methods presented above because of the imperfect conservation of the binding elements between mouse and human.
